# From Schizophrenia Genetics to Disease Biology: Harnessing New Concepts and Technologies

**DOI:** 10.20900/jpbs.20190014

**Published:** 2019-09-19

**Authors:** Jubao Duan, Alan R. Sanders, Pablo V. Gejman

**Affiliations:** 1 Center for Psychiatric Genetics, NorthShore University HealthSystem, Evanston, IL 60201, USA; 2 Department of Psychiatry and Behavioral Neurosciences, The University of Chicago, Chicago, IL 60637, USA

**Keywords:** schizophrenia, genetics, genome-wide association study, functional genomics, epigenomics, chromatin modification, model organism, human induced pluripotent stem cells (hiPSCs), genome editing

## Abstract

Schizophrenia (SZ) is a severe mental disorder afflicting around 1% of the population. It is highly heritable but with complex genetics. Recent research has unraveled a plethora of risk loci for SZ. Accordingly, our conceptual understanding of SZ genetics has been rapidly evolving, from oligogenic models towards polygenic or even omnigenic models. A pressing challenge to the field, however, is the translation of the many genetic findings of SZ into disease biology insights leading to more effective treatments. Bridging this gap requires the integration of genetic findings and functional genomics using appropriate cellular models. Harnessing new technologies, such as the development of human induced pluripotent stem cells (hiPSC) and the CRISPR/Cas-based genome/epigenome editing approach are expected to change our understanding of SZ disease biology to a fundamentally higher level. Here, we discuss some new developments.

## INTRODUCTION

Schizophrenia (SZ) is a devastating mental disorder that afflicts ~1% of the population worldwide. The core features of SZ include both positive symptoms (delusions, hallucinations, and disordered thinking) and negative symptoms (social withdrawal, lack of motivation, and poverty of speech), as well as some cognitive impairments [1]. SZ, like other psychiatric disorders, presents significant phenotypic heterogeneity [1]. Despite the recent successes in better understanding genetic risk for SZ, the causal molecular mechanisms underlying these genetic findings and their contributions to SZ pathophysiology remain largely unresolved. Furthermore, the development of most antipsychotic drugs was guided by concepts originated before the current wave of genetic findings [2]. A more sophisticated understanding of the molecular pathophysiology of SZ is critical for developing more effective therapeutic targets.

**Figure 1.**
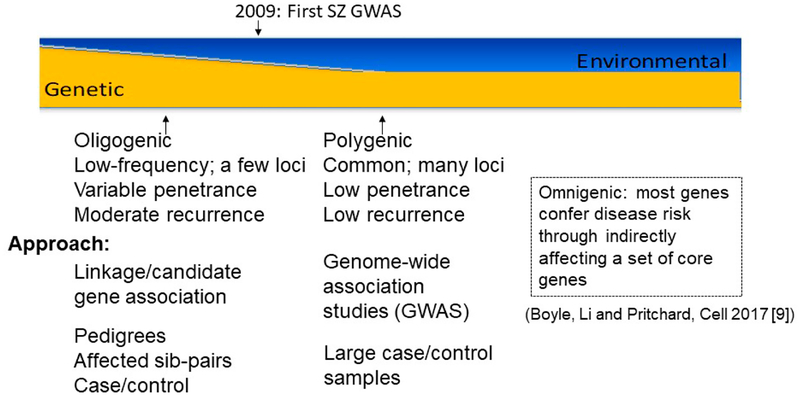
Evolving understanding of schizophrenia genetics from oligogenic to polygenic and omnigenic models.

The breakthroughs from recent large-scale genome-wide association studies (GWAS) [3–8] provide an unprecedented opportunity to gain biological insights and identify novel drug targets. From the earlier stage of genetic linkage studies designed to identify only a handful of highly penetrant disease genes under an oligogenic model to the most recent mega GWAS, our understanding of SZ genetics has experienced a dramatic change (Figure 1). We now estimate a landscape of hundreds of disease loci spanning thousands of disease genes, each contributing a small population risk. This model has been extended to a new paradigm, the omnigenic model, which proposes that most or all genes that are expressed in a disease relevant cell type may confer SZ susceptibility through perhaps interacting with a set of core disease risk genes [9]. Furthermore, a relatively still modest number of large and rare copy number variants (CNVs) with larger effect on disease susceptibility than common GWAS risk variants have also been identified [10–13]. Moreover, large-scale whole exome (WES) or genome (WGS) sequencing studies conducted on increasingly large (and more diverse) samples are anticipated in the near future to identify many new, rare SZ risk variants. Continuously larger samples are available from national biobanks, of which the UK Biobank is a stellar example of productivity [14,15]. Environmental factors (some of which can in turn be modified by genetic phenotypes of parents and children), such as level of stress, viral infections, nutritional deficits, and family cognitive characteristics may add or subtract risk, independently, or through interaction with genetic factors to increase susceptibility risk in more complex models, some including epigenomic components.

The polygenic theory of SZ proposed by Gottesman and Shields [16] is consistent with the overall GWAS results, but before the GWAS “era” many in our field hoped for SZ to be explained by more simple models, which was reflected in inadequate study designs. For example, SZ candidate gene studies have led the field to many non-replications [17–19]. Furthermore, GWAS work was also instrumental to a better characterization of rare CNVs and allowed the deconstruction of disease pleiotropy. The majority of the SZ-associated CNVs are also associated with multiple other neurodevelopmental and neuropsychiatric conditions, such as autism, intellectual disability and epilepsy (see review [20]). Here, we briefly review aspects of the historical journey from genetic linkage studies to GWAS, mainly focusing on recent developments of common-variants-common-disease (CVCD) model. We highlight the novel biological insights derived from by functional genomics approaches that help connect genetic findings with disease biology. Given that most common GWAS risk variants of SZ are located in the noncoding parts of the genome likely to regulate gene expression, our review of functional genetics of SZ will focus on the transcriptomic analyses and chromatin modification in SZ, and provide a glimpse of novel research strategies that may help better understand novel disease biology of SZ.

## PRE-GWAS GENETIC LINKAGE AND ASSOCIATION STUDIES OF SZ

Genetic linkage studies identify large genetic regions (in cM scale) that may co-segregate with disease risk in pedigrees or affected sib-pairs (ASPs). The assumption of this approach for SZ at that time was that there were only a handful disease risk genes of relatively large effect size, but with common population frequency [21]. The linkage studies of SZ have been extensively reviewed previously [22–25], and we only highlight here some late-stage linkage findings from meta-analyses and in large samples. In 2002–2003, meta-analyses of linkage studies from genome scans of samples of European ancestry (EA) did find supporting evidence for susceptibility loci on 13q and 22q for bipolar disorder, and on multiple chromosomal regions for SZ [26,27]. Later on, the Molecular Genetics of SZ (MGS) collaboration detected two chromosomal regions with suggestive evidence of linkage on chromosomes 8p23.3-p12 and 11p11.2-q22.3 in 409 pedigrees with SZ—263 of EA and 146 of African American (AA) ancestry [28]. Overall, the SZ linkage studies have nominated susceptibility loci spanning large genomic regions on many chromosomes, suggesting a possibly much larger number of risk genes than one would expect from the oligogenic inheritance model. Furthermore, these linkage studies did point to the locations of some specific SZ risk genes identified later in the GWAS [3–8], e.g., *CUB* and *Sushi Multiple Domains 1* (*CSMD1*) at 8p, one of the most consistently observed regions in SZ linkage scans [28].

Like linkage studies, candidate gene association studies of SZ were also a major effort in the pre-GWAS era. The selected candidates were often either physically located in some suggestive SZ linkage regions (positional candidates) or known to have some functions possibly related to SZ (etiological candidates). These candidate gene studies often used very underpowered samples (either family-based or case-control) and unrealistic genetic models. In addition, because of the limited throughput of genotyping technology and the lack of information on linkage disequilibrium (LD) relationships between genetic markers at that time, these pre-GWAS candidate gene studies often analyzed a small number of genetic markers (mostly single nucleotide polymorphisms-SNPs). Thus, most candidate gene association studies reported inconsistent findings [29]. Although these pre-GWAS linkage and association studies did not lead to the identification of any specific SZ susceptibility loci or genes, the non-replications confronting the field highlighted the complexities of genomics in general, and of SZ genetics in particular. Some of the samples used in these pre-GWAS studies also contributed to build the foundation of large study cohorts that were essential to the later success of SZ GWAS, but the main GWAS samples in the US were built from the start for more ambitious, and ultimately more productive, genome-wide association testing.

## GWAS OF COMMON VARIANTS PRODUCE REPLICABLE FINDINGS OF SZ GENETICS AND UNCOVER POLYGENICITY AND PLEIOTROPY

The success of SZ GWAS [3–8] was enabled by high throughput genotyping technology that allowed the assay of millions of common variations, and most importantly, in sufficiently large research samples. The combined analysis of three GWAS samples in 2009 (MGS, ISC, SGENE; 8008 cases, 19,077 controls) uncovered a genome-wide significant locus on chromosome 6p22.1 (the best SNP with a *P* = 9.54 × 10^−9^) spanning the extended Major Histocompatibility Complex (MHC) region [3]. The landmark GWAS finding with MHC highlighted the importance of sharing data to increase statistical power, laying the scientific foundation and framework for the Psychiatric Genomics Consortium (PGC) to perform GWAS meta-analyses of SZ and other major psychiatric disorders. In 2014, with 36,989 SZ cases and 113,075 controls, the PGC (Phase 2) GWAS study of SZ identified 108 independent risk loci, spanning hundreds of genes [8] and the number of associated loci continues to grow [30], of which MHC remains to be the most strongly associated locus. Importantly, the GWAS findings supported a long-standing (although little appreciated for many years) polygenic hypothesis of SZ [16]. The International SZ Consortium (ISC) GWAS dataset first empirically tested this hypothesis using aggregate risk scores defined by sets of common variants with small effects (also known as a polygenic risk score, PRS) [4]. Since then, polygenic risk prediction continues to develop although it remains for now in the research, not the clinical, arena (reviewed in [31]).

Analyzing GWAS data sets of SZ and other common disorders also reveals substantial genetic pleiotropy (*i.e*., the same genetic locus contributing to susceptibility to multiple phenotypes) between SZ and other disorders. An analysis of 25 brain disorder GWAS datasets (265,218 patients and 784,643 controls) found that major psychiatric disorders substantially shared common variant risks between disorders and a number of brain phenotypes, but not with neurological disorders such as Alzheimer’s disease [32]. A systematic analysis of genetic correlations between SZ and 172 medical, psychiatric, personality, and metabolomic phenotypes identified the strongest genetic correlation with bipolar disorder [33]. A joint analysis of genetic data consisting of 53,555 cases (20,129 bipolar disorder, 33,426 SZ) and 54,065 controls further identified 114 genome-wide significant loci implicating synaptic and neuronal pathways shared between the two disorders [34]. These observations are consistent with the known comorbidity and shared symptoms between these psychiatric disorders. As others have discussed [35], although they have distinct clinical diagnostic criteria, a shared or convergent biological causal pathway may have led to some common clinical symptoms across these psychiatric disorders.

In the presence of pleiotropic effects, it is challenging to fully distinguish genetic risk factors specific to one disorder from those from other disorders. Assuming the pleiotropic factors across disorders are true genetic associations, integration with data from functional genetic dimensions such as network-level multi-omics data, molecular and cellular phenotypes from iPSC-derived cellular models (see below), and physiological measurements at brain circuitry levels, hold the promise to contribute to disentangling disorder-specific signatures.

## IMPLICATIONS OF RARE SZ RISK VARIANTS IDENTIFIED BY CNV AND WHOLE EXOME SEQUENCING STUDIES

The full spectrum of SZ risk variants also includes rare and relatively highly penetrant ones. A number of rare and long (>100 kb, usually spanning multiple genes) genomic segments of duplications or deletions, *i.e*., CNVs, were found strongly associated with SZ with a much larger effect size (odds ratios 2–70) than common SNPs [10–13], including 1q21.1, 2p16.3 (*NRXN1*), 3q29, 7q11.2, 15q13.3, distal 16p11.2, proximal 16p11.2, and 22q11.2. Of these, the 22q11.2 deletion was well known to cause the 22q11.2 deletion syndrome (22q11DS), of which ~30% of the carriers develop SZ [36]. There was also a global enrichment of CNV burden in cases (21,094 SZ cases and 20,227 controls) [13]; however, the low odds ratio (~1.1) suggested that most of these CNVs did not contribute large effects for SZ risk. Furthermore, the rare CNVs can only explain 0.85% of the variance in SCZ liability [13]. On the other hand, due to their relatively large effect sizes, these rare CNVs provide a model where the disease relevance of cellular phenotypes is easier to interpret.

On an exome sequencing front, a recent meta-analysis of WES from 4133 SZ cases and 9,274 controls, together with *de novo* mutations (DNMs) in 1077 families, only identified one single gene, *SETD1A* that encodes an H3K4 tri-methyltransferase, harboring rare deleterious mutations (stop-gain or frame-shifting) that were collectively associated with SZ with genome-wide significance [37]. The currently available sequencing data thus does not seem to suggest that rare risk variants can explain much of the SZ risk variance. However, with future WES or WGS of SZ in much larger samples, the importance of the contribution of rare variants to the polygenic risk spectrum of SZ may evolve. With sufficiently large numbers of both common and rare variants, Fisher’s additive genetic risk model of large numbers of variants of infinitesimal effect seems to give a good approximation to the total heritability of complex disorders [38]. Using the largest WGS sample of 21,620 unrelated individuals from the Trans-Omics for Precision Medicine (TOPMed) program, it has been recently shown that rare variants, particularly those in low LD with nearby SNPs, combined with the common GWAS variants, can explain the full genetic heritability estimated from pedigrees for complex traits like height and BMI [39].

It is noteworthy that although rare coding SNPs with low LD predicted to affect protein structure or function seem to individually explain much larger risk variance, other types of rare variants including synonymous coding and regulatory non-coding variants do explain a large share of disease risk [39]. This highlights the importance of functional interpretation of noncoding regulatory variants (see below sections) identified in WGS and coding synonymous variants identified in WES. Current WES efforts have been focusing on DNA variants that predict a structural change in a protein, *i.e*., loss-of-function (LoF) variants or deleterious missense variants. However, there is also evidence that synonymous variants (or “silent” mutations), *i.e*., those do not change protein sequences, may be functional through changing mRNA stability or preferred amino acid codon, affecting gene expression at various levels [40]. In this regard, we have previously shown that synonymous SNPs in *dopamine D2 receptor* (*DRD2*), a gene of psychiatric importance, can affect its mRNA stability and receptor protein synthesis [41–43]. Predicting functional synonymous variants identified from WES may thus open a very large potential source of DNA variation that could be relevant to common disorders, but this mechanism has remained largely unexplored until now.

With both rare and common risk variants as parts of the SZ risk spectrum, an intriguing question is whether SZ rare risk variants etiologically overlap with common risk variants. Joint analyses of SZ-associated CNVs and common GWAS risk variants seemed to support an additive model of both types of genetic risk factors. Compared with controls, SZ subjects who carried the SZ-associated CNVs possessed an excess burden of common GWAS risk alleles [44]. While carrying higher PRS than controls, CNV-carrying SZ cases seem to have lower PRS than SZ cases without risk CNVs [45]. Therefore, both common and rare genetic risk factors constitute a complex genetic architecture of SZ, interactively influencing disease liability. For most rare risk variants, a polygenic common risk genetic background may provide a necessary threshold to enable their disease penetrance. This implies that modeling the functional effects of SZ rare risk variants in cellular models may need to be on genetic backgrounds with sufficient polygenic risk of SZ.

## MAIN BIOLOGICAL INSIGHTS INFORMED BY GENETIC FINDINGS

From knowing very little about the chromosomal locations of disease risk loci a decade ago, over 100 replicable SZ risk loci have been reported by now. These genetic findings offer us an unprecedented opportunity to understand disease biology.

### Immune Hypothesis—MHC, C4A and Synapse Pruning

The MHC region, containing a high density of immune-related genes, shows the strongest association in SZ GWAS, supporting a long-standing immune hypothesis of SZ [46]. A Danish registry study also concluded that autoimmune disorders increase the risk for SZ [47]. The MHC region has also been implicated by GWAS in multiple common immune diseases, including type 1 diabetes, multiple sclerosis, Crohn’s disease, and rheumatoid arthritis (see review [48]). Even without considering the MHC region, SZ-GWAS-implicated genes are also enriched in key immune processes such as TGF-β signaling, B-cell activation, and T-cell activation [8]. Cytokines play roles in cytotoxicity and apoptosis, and influence neurotransmitter systems [49,50] that are implicated in the pathophysiology of SZ. Large transcriptomic studies have also strongly implicated immune system genes as being enriched for differential expressed in SZ [51–53].

Recently, common alleles of *complement component 4A* (*C4A*) have been proposed to underlie some of the GWAS signal at the MHC region [54], in itself, a locus of small effect. Higher copy number of *C4A* has been reported as associated with increased expression in SZ postmortem brains [54]. Because of the known pivotal role of complement signaling in microglia-mediated synapse pruning in the developing mouse brain, *C4A* was postulated to be important in the synapse pruning process of human brain during late adolescence and early adulthood [55], a hypothesis also proposed for other psychiatric disorders (for example, see [56]). Most recently, this hypothesis was tested in an iPSC-derived *in vitro* model of microglia-mediated synapse engulfment, showing that *C4A* is associated with increased neuronal complement deposition and synapse uptake in SZ-derived microglia and reduced neuronal synaptic proteins [57]. Pending replication of its multiple aspects, the *C4A* hypothesis of SZ holds the promise of unravelling new pathophysiological mechanisms.

Although microglia are not a cell type that is so genetically relevant to SZ (*i.e*., enriched for genetic risk factors of SZ)[58], the *C4A* finding highlights the importance of microglia in synapse pruning in SZ. Microglia are a residual immune cell type in the brain, mediating brain immune responses, facilitating brain maturation through synapse pruning, and clearing up cellular debris and dead neurons via phagocytosis. It has been shown that classical antipsychotics affect microglial cells and astrocytes in the central nervous system partly through the modulation of the expression of *cyclo-oxygenase-2* (*COX-2*) [59,60]. There is also growing evidence from clinical studies with COX-2 inhibitors that points to favorable effects of anti-inflammatory therapy in SZ [59,60]. Most recently, type I interferon was shown to activate the synapse pruning function of microglia in lupus-prone mice, suggesting a mechanism underlying the prevalent neuropsychiatric conditions in patients with systemic lupus erythematosus [61].

### Dopaminergic Neurotransmission

The hyperdopaminergic hypothesis of SZ rests on the observation that psychotogenic stimulants such as methamphetamines lead to elevated brain dopamine (DA) levels and can cause psychosis [2,62–69] and that most commonly used antipsychotic drugs that were developed five decades ago for treating the positive symptoms of SZ target DRD2. Thus, the SZ GWAS association at the *DRD2* locus appears to be congruent with the idea that leveraging GWAS hits, despite of their small effect sizes could lead to the identification of more effective new drug targets for treating SZ.

However, DA has multiple different mechanisms of action. With B-cell transformed lymphoblastoid cell lines (LCLs) as a cellular model, we carried out DA cellular stimulation and analyzed the DA-induced transcriptomic differences between 514 SZ cases and 690 controls [51,70]. We found that the set of genes differentially expressed in DA-stimulated LCLs between SZ cases and controls were enriched for genes related to immune processes and apoptosis as well as mitochondrial oxidative phosphorylation, suggesting that DA may play a role in SZ pathogenesis through modulating those systems [51,70]. Moreover, the DA-induced SZ-associated differentially expressed genes also showed a trend towards enrichment of genes near genome-wide significant SZ loci and with genes spanned by SZ-associated CNVs [51,70], suggesting a potential link between the implicated DA pathway and other genomic risk factors. However, LCLs as a cellular model has its obvious limitations, *i.e*., further-removed from brain that is presumably the most relevant tissue for SZ, and a future DA perturbation experiment in a more disease relevant iPSC-derived neural model may thus shed light on how DA and *DRD2* function in conferring SZ risk. Finally, it is noteworthy that the putative causal variant(s)/gene(s) at the *DRD2* locus, like for most other SZ risk loci, have not been established. It thus remains possible that some other genes such as *neural cell adhesion molecule 1* (*NCAM1*) that is ~180 kb away from *DRD2*, rather than *DRD2* itself, may be the actual “causal” gene at this GWAS locus.

### Glutamatergic Neurotransmission

Both SZ GWAS and WES studies lend stronger support to abnormal glutamatergic neurotransmission than to the dopaminergic hypothesis in SZ. More GWAS-implicated genes seem to be involved in glutamatergic neurotransmission than other neurotransmitter systems. Out of 108 SZ risk loci, at least eight contained genes related to synapses or excitatory neurotransmission (*CLCN3*, *GRIA1*, *ELFN1*, *CALHM1*, *CACNA1C*, *GRIN2A*, *SRR*, and *CACNA1I*). Collectively, gene set analysis of the GWAS-implicated genes of three major psychiatric disorders (SZ, major depression, and bipolar disorder) identified glutamatergic synaptic neurotransmission as one of the most enriched gene pathways [71].

WES [72,73] and CNV studies [12] also strongly support the role of abnormal synaptic plasticity and glutamatergic neurotransmission in SZ. Exome sequencing in 2,536 SZ cases and 2,543 controls suggest that rare disruptive mutations were enriched in gene sets related to voltage-gated calcium ion channel and the signalling complex formed by the activity-regulated cytoskeleton-associated scaffold protein (ARC) of the postsynaptic density [72]. Exome sequencing also identified that DNMs were overrepresented in glutamatergic postsynaptic proteins comprising ARC protein and *N*-methyl-D-aspartate receptor (NMDAR) complexes [73]. For CNVs, there is an increased burden of the largest CNVs (>500 kb) in genes present in the postsynaptic density [12]. Gene pathway analyses of SZ-associated CNVs also supported the enrichment of genes related to synaptic functional and neurobehavioral phenotypes in the mouse [13].

These genetic findings converge with previous pathophysiological knowledge of the abnormalities of synaptic neurotransmission in SZ, mostly from post-mortem brain studies. SZ patients show a reduction of cortical grey matter volume and thickness, as well as reduced functional cortical connectivity [74–77]. Reductions in dendritic spine density on cortical pyramidal neurons are thought to directly contribute to these abnormalities [74,75,78,79]. Overall, these post-mortem studies are heterogeneous, often with small sample sizes. A recent study aiming to link observed abnormal glutamatergic neurotransmission to specific SZ GWAS genes in a zebrafish model carrying mutant orthologs of 132 SZ GWAS genes reinforced the possible contributions of genes and pathways related to glutamatergic neurotransmission (*gria1*, *grin2a*, *elfn1*, *clcn3*), calcium channel function (*cacna1c*, *cacn2b*), neurodevelopment (*bcl11b*, *foxg1*, *mir137*, *tcf4*, *gpm6a*), and complement regulation (*csmd1*) to SZ risk [80]. Further study on the relevance of each of these individual GWAS- or WES-implicated SZ risk genes or pathways to SZ in disease-relevant human cell types and animal models might lead to deeper insight into understanding molecular and cellular causal mechanisms of these genetic findings related to glutamatergic neurotransmission.

### Abnormal Chromatin Modification as a Plausible Novel Causal Mechanism

Both GWAS and WES provide supporting evidence for the involvement of chromatin modifications in SZ. Common SNPs at *Bromodomain containing 1* (*BRD1*) on 22q13.33 approached genome-wide significance (*P*  =  3.31  ×  10^−7^) in the PGC2 SZ GWAS [8] and reached genome-wide significance by using an Empirical Bayes statistical approach [81]. Gene set enrichment analyses of common SZ risk variants from GWAS, CNVs, and rare risk variants from sequencing have strongly suggested abnormal chromatin remodeling and modification to be among the most relevant disease mechanisms for SZ. By analyzing GWAS-implicated genes in 60,000 participants for three major psychiatric disorders (SZ, major depression, and bipolar disorder), PGC identified the histone methylation process as the most enriched gene pathway [71].

Consistently, analyzing genes harboring rare SZ-risk variants identified in WES also strongly supports the etiological role of chromatin remodeling and histone modifications in SZ. DNMs identified in 57 trios with sporadic or familial SZ were found specifically enriched in genes (*CHD8*, *MECP2*, and *HUWE1*) involved in epigenetic regulation (DNA methylation and chromatin structure) of transcription, suggesting the importance of chromatin modification as a risk mechanism for SZ [82]. Collectively, nonsense DNMs were significantly enriched among a set of 419 genes characterized by domains highly specific to chromatin modification and more importantly, there was an overall significant over-representation of genes involved in chromatin organization [82]. WES of SZ in much larger datasets also supported the importance of chromatin modification in SZ pathogenesis [83], and a meta-analysis of WES from 4,133 SZ cases and 9274 controls, together with DNMs in 1,077 family trios and CNVs from 6882 cases and 11,255 controls, further concluded that several gene sets enriched for rare coding variants were all related to chromatin modification and organization [37]. With the currently available WES datasets, the only individual gene that has rare LoF variants (in aggregate) associated with SZ at genome-wide significance is *SETD1A* that encodes H3K4 trimethyltransferase [84,85]. Given that H3K4me3 is a key epigenomic mark for active promoters for gene expression, a dysfunctional *SETD1A* is expected to cause haploinsufficiency of genes essential for many biological processes. A recent study found *SETD1A* can affect H3K4me3 levels and β-catenin expression, which may be important for regulating stem cell self-renewal, neural progenitor cell (NPC) proliferation, and neurogenesis [86]. Given the relatively high penetrance of LoF mutations in *SETD1A*, identifying the specific targets of *SETD1A* in neuronal cells and the brain-specific roles of *SETD1A* may help establish a causal link between chromatin signaling and SZ pathogenesis.

As for CNVs, given that large chromosomal deletions and duplications are known to affect specific 3D chromatin topologically associating domains (TADs) [87,88], it is conceivable that some SZ-associated CNVs may confer disease risk through impairing local or distal chromatin architecture, and subsequently altering expression of a cluster of genes within or outside the CNV region. Indeed, observations on the SZ-associated 22q11.2 deletion strongly suggest the possible involvement of chromatin modifications and remodeling in SZ. 22q11.2 deletion-carriers vs. controls exhibited differential genome-wide chromatin interaction profiles, with some long-range or *trans*- (*i.e*., in other chromosomes) chromatin interactions anchored with the deletion region observed in controls but absent in deletion-carriers [89]. Consistent with the observed long-range chromatin interaction mediated by the 22q11.2 deletion region (of note, the locus conveying the highest risk for SZ), a recent transcriptomic study of SZ-associated rare CNVs in LCLs identified abundant gene expression changes (~500 genes) outside of the 22q11.2 deletion region, and these genes were highly enriched with gene ontology (GO) terms related to chromatin modification and organization [90]. The broad transcriptomic effects of the 22q11.2 deletion may be attributed to the CNV-induced changes of 3D chromatin TADs [87,88]. Alternatively, it may be also due to some specific genes within the 22q11.2 region, e.g., the histone chaperone, *HIRA*, which can interact with *SETD1A*. Therefore, the 22q11.2 deletion (and possibly other large SZ-associated CNVs) may have widespread effects on chromatin organization, which may contribute to the high penetrance of the CNV as well as its inherent phenotypic variability.

By integrating SZ GWAS data and human brain and/or neuronal transcriptomic data, our recent gene network analyses also suggest the possible pathogenic role of chromatin modifications [91]. Using RNA-Seq data on postmortem dorsolateral prefrontal cortex (DLPFC) from SZ patients and control subjects, we deconvolved the transcriptional network to identify SZ-associated master regulators (MRs) that mediate expression of a large body of target genes. Two top candidate SZ-associated MRs, *TCF4* (a leading SZ risk locus implicated by SZ GWAS) and *HDAC9*, may both function through affecting histone modifications: approximately 77% of the *TCF4*-binding sites overlapped with the H3K27ac marks in the genome [92], while *HDAC9* itself is a histone deacetylase that potentiates hippocampal-dependent memory and synaptic plasticity [93,94]. These results highlight the relevance of chromatin modification to SZ pathogenesis.

Altogether, data from SZ GWAS, SZ-associated brain transcriptomic gene networks, and SZ-associated rare variants (SNPs and CNVs) strongly suggest that chromatin modification may act as an epigenetic regulatory hub in certain core SZ gene networks. Of all the plausible biological disease mechanisms informed by SZ genetic findings, chromatin modification represents a novel and powerful one. This is because chromatin modification presumably affects many genes in many cell types across different developmental stages. The importance of such an epigenomic feature in conferring SZ risk is in accordance with the long-standing hypothesis that environmental risks for SZ occur at critical periods early in development, such as maternal malnutrition seen in the Dutch Hunger Winter and Chinese famine studies [95]. The early developmental stage is a time when the epigenome is particularly labile and is correlated with rapid cell replication, differentiation, and tissue specification. A better understanding of the SZ-associated differential chromatin modifications and their genetic control will help establish the causal link between abnormal chromatin modification and cell type or developmental stage-specific gene expression changes that lead to disease phenotypic manifestation.

## CHROMATIN MODIFICATIONS AND GENETIC CONTROL IN SZ

Chromatin consists of units of nucleosomes with segments of double stranded DNAs wrapping around histones. Chromatin states and modifications strongly influence gene expression. We discuss below the main chromatin modifications relevant to SZ, which include DNA methylation, histone acetylation, and histone methylation.

### DNA Methylation

Methylated DNA regions are often associated with repressed transcription, likely mediated by weakening transcription factor (TF)-binding and/or altering the chromatin state. DNA methylation influences transcriptional regulation, genomic stability, chromatin structure modulation, and development [96]. As one of the major epigenetic modifications, the role of DNA methylation has been extensively studied in peripheral blood [97] and postmortem brain tissues [98] for neuropsychiatric disorders, including SZ. Compared to studying blood cells, a genome-wide methylation study in postmortem DLPFC (191 SZ cases and 335 controls) presented more biologically meaningful supportive evidence for aberrant DNA methylation in SZ [98]. A total of 2104 CpG sites showed significant differential methylation, and genes flanking these SZ-associated loci were significantly enriched for embryonic development, cell fate commitment, and nervous system differentiation. These large-scale genome-wide methylation studies in SZ suggest a possible role of aberrant DNA methylation in the disease etiology.

The differences of DNA methylation between SZ patients and controls may be attributed to both non-genetic factors and an individual’s genetic makeup. Abundant genetic variants are found associated with DNA methylation, *i.e*., methylation quantitative trait loci (meQTLs), which in the brain have suggested a direct mechanistic link to disease biology for some specific disease risk variants [99,100]. For instance, Jaffe and colleagues identified 4.1 million significant meQTLs and found that ~60% of genome-wide significant SZ loci had a risk or proxy SNP that was a meQTL. In another study of 166 developing human fetal brains, Hannon and colleagues identified abundant meQTLs that were highly enriched (~4-fold) for genome-wide significant SZ risk variants [100]. Altogether, the differential DNA methylation between SZ cases and controls and the enrichment of brain meQTLs for SZ GWAS risk variants support the possible pathophysiological relevance of DNA methylation to SZ. However, because of SNP LD, an meQTL also associated with SZ may not be the causal SNP itself. The interpretation of the mechanistic link between DNA methylation and causal SZ variants thus remains a challenge.

### Histone Methylation

Histone methylation mostly occurs at lysine (K) positions of H3 or H4, resulting in various types of histone marks with diverse functions that can cause transcriptional activation or repression, depending on the positions of the methylated K residue and the number of added methyl groups. Abnormal histone methylation of some specific loci in SZ has been demonstrated in human post-mortem brains [101,102]. Furthermore, SZ-associated H3K4 methylation changes of hundreds of loci were reported in immature neurons derived from olfactory epithelium cells of a very small number of SZ cases/controls (*n* = 4) [103]. It remains to be tested whether there are SZ-associated differential histone methylations at a genome-wide scale in relatively large and disease-relevant cells/tissues.

The strongest supporting evidence for the involvement of histone methylation in SZ is the association of rare LoF mutations in *SETD1A* in aggregate with SZ [84,85], a gene that encodes part of a H3K4 trimethyltransferase complex. As discussed above, the SZ-associated heterozygous LoF mutations in *SETD1A* are expected to cause ~50% reduction of *SETD1A* and impair H3K4 methylation. However, mutant mice carrying a heterozygous LoF mutation in *Setd1a*, despite exhibiting alterations in axonal branching and working memory deficits, did not show significant alteration of global brain H3K4me3 [104]. This suggests that the heterozygous mutant-associated biochemical change of histone methylation may have been compensated by other homologous genes such as *SETD1B*; alternatively, *SETD1A* may function through other pathways in brain. Future study on how *SETD1A* can affect SZ-relevant cellular phenotypes in human neuronal models may help determine whether H3K4me3 is impaired by SZ-associated *SETD1A* mutations during early neurodevelopmental stages.

### Histone Acetylation and Deacetylation

Histone acetylation and deacetylation are modulated by histone acetyltransferases (HATs) and histone deacetylases (HDACs), respectively. The former is associated with a more open chromatin and active transcription, while the latter often leads to a more condensed chromatin and transcriptional repression. Earlier studies of histone acetylation in SZ were performed in both blood cells and post-mortem brain tissue from very small samples. The baseline levels of H3 K9/K14 acetylation in blood cells were lower in SZ cases than in controls [105]. Consistent results were also found in post-mortem brain tissue, showing that the histone H3 K9/K14 at the promoters of some SZ candidate genes were hypoacetylated in SZ [106]. Although there has been no systematic investigation of abnormal histone acetylation in a large number of SZ post-mortem brains, a recent genome-wide histone acetylation study on autism spectrum disorder (ASD)[107] may provide some insights into SZ, given the genetic correlation between SZ and ASD [32]. Profiling the histone acetylation, H3K27ac (mark of active enhancers), in 257 postmortem brain samples of ASD patients and matched controls, identified a common acetylome signature for >5000 *cis*-regulatory elements in the prefrontal and temporal cortex [107]. Furthermore, some of the brain histone acetylation quantitative trait loci (haQTLs) overlap with putative causal variants for SZ and ASD (e.g., rs4765905 in *CACNA1* and rs8054791 in *GRIN2A*) [107]. Notably, ~77% of the binding sites of *TCF4*, a TF encoded by a leading SZ GWAS risk gene, overlap with the H3K27ac region [92], suggesting possible convergence between *TCF4* and a seemingly non-specific histone modification pathway.

### 2D and High-Order (3D) Chromatin Structures

Some parts of the mammalian genome are open with loosely organized euchromatin, which is associated with active transcription. Open chromatin is correlated with epigenomic histone modifications associated with active enhancers and promoters (e.g., H3K4me1 and H3K4me3) [108–112], thus a versatile index of regulatory sequence elements. Open chromatin has been recently studied for its relevance to SZ in both human brains and neurons derived from hiPSC. Fullard and colleagues first applied ATAC-seq (Assay for Transposase-Accessible Chromatin using sequencing) to map open chromatin region (OCR) profiles in sorted neuronal and non-neuronal nuclei isolated from frozen postmortem human brain [113]. They found enrichment for SZ risk genes in both neuronal and non-neuronal OCRs, with neuronal OCRs showing higher enrichment (2-fold *vs* 1.6-fold). With hiPSC-derived relatively purer excitatory neurons as a cellular model, we have also recently mapped global OCRs by ATAC-seq and found that neuronal OCRs are enriched for GWAS-implicated SZ risk variants [114]. Co-localization of SZ GWAS variants with neuronal OCRs can narrow down putatively functional SZ risk variants to ~100 SNPs that are within neuronal OCRs that flank TF binding sites [114]. At a leading SZ risk locus spanning *MIR137*, we showed that the risk allele of common GWAS risk SNP rs1198588 was associated with altered *MIR137* promoter chromatin openness, reduced *MIR137* expression, and accelerated neuronal maturation [114]. These studies thus suggest that noncoding GWAS risk variants in OCRs may impact the neurodevelopmental aspect of SZ.

Because the mammalian genome is a 3-dimensional (3D) structure, it is expected that the physically interacting chromatin domains can coordinate cellular gene expression programs [115]. The 3D chromatin structure encodes the functional relationship between a *cis*-regulatory DNA sequence element and its distal target genes. The relevance of 3D chromatin structure to SZ has been recently shown by studying how brain 3D genome mediates the effect of putatively functional risk variants and their target genes in SZ GWAS loci. Through analyzing the genome-wide chromatin conformation capture by high throughput sequencing approach (*i.e*., Hi-C) in developing human cerebral cortex, Won and colleagues found that many SZ GWAS risk SNPs interact with their flanking genes at a chromatin level and frequently with genes not adjacent to the index SZ GWAS risk SNPs, or in LD with them [116]. It is noteworthy that the SZ GWAS risk SNPs interacting with the promoter of *DRD2* are also connected with enhancers/promoters of *GRIA1* and *GRIN2A*, the latter supporting the important role of abnormal glutamatergic neurotransmission in SZ pathophysiology [7,8,12,72,73]. Therefore, high-order chromatin structure may play an important role in modulating temporal and spatial gene expression relevant to SZ. A comprehensive map of 3D chromatin structures in human brains and in different subtypes of neuronal cells may further shed light on the molecular causal mechanisms of SZ in relationship to high-order chromatin structure.

## HIPSC-DERIVED NEURONS AND BRAIN ORGANOIDS AS A MODEL FOR STUDYING SZ DISEASE BIOLOGY

For biological follow up of genetic findings, because of the inherent limitation on drawing causal inferences about psychiatric disease mechanisms, it is critical to clearly define the research question, carefully select the most appropriate experimental system, and rigorously design a well-powered study (see guidelines in a recent review [117]). Tractable and reproducible experimental models relevant to SZ are pivotal for bridging the gap between genetic findings and disease biology. Each experimental model has its own pros and cons. Somatic cells such as fibroblasts or blood cells from patients or healthy controls can be reprogramed into stem cells (hiPSC) and then re-differentiated into different subtypes of neuronal cells relevant to brain disorders. Such hiPSCs-derived neuronal cells are a promising cellular model for neuropsychiatric disorders [118,119], offering an excellent alternative to the use of human post-mortem brains. First, compared to human brains and the emerging brain organoid model [120,121], iPSC-derived neurons are relatively homogeneous [119,122]. For instance, the forced exogenous expression of NGN2 gives rise to ~100% excitatory neurons in about 4 weeks [123,124]. Second, an hiPSC model enables studying molecular and cellular phenotypic changes relevant to SZ disease biology (as discussed above) in a temporal and cell type-specific manner. Third, compared to postmortem brain, which is well-known to be confounded by tissue variability and environmental factors [125], hiPSC differentiation into neurons can be better controlled, and thus the data may be more reproducible. Lastly, compared to postmortem brain, hiPSC models are amenable to genetic modification or epigenomic perturbation, which is important for studying the functional impacts of disease-relevant chromatin dysregulation. Therefore, when combined with genome editing, hiPSC-derived neurons represent a powerful cellular model for understanding disease biology underlying SZ genetic findings [126].

What types of iPSC-derived cells are needed for biological follow up largely depends on the specific genetic variants of interest or specific questions to be addressed. Multiple subtypes of neuronal cells can be derived from iPSC to model SZ-relevant molecular and cellular phenotypes based on the aforementioned genetically-informed SZ disease biology. By using different combinations of growth factors and small molecules in culture media, hiPSCs can be efficiently differentiated into midbrain dopaminergic neurons [127], cortical glutamatergic neurons [128], GABAergic inhibitory interneurons [129–131], and microglia [132]. As an alternative to using defined growth factors in culture, forced expression of exogenous TFs can rapidly differentiate hiPSCs into functional subtypes of neurons, for instance, forced expression of NEUROD1 or NEUROG2 rapidly induces the differentiation of iPSCs into excitatory neurons [123,124], while forced expression of ASCL1 and DLX2 induces the differentiation of iPSCs into GABAergic inhibitory interneurons [133]. How to derive these cell types in a more efficient way and into other types of brain cells such as oligodendrocytes in 2D culture is a rapidly evolving area. Compared to 2D-culture of hiPSC-derived neural cells, iPSC-derived brain organoids or spheroids are recently becoming a more attractive and promising model that can better recapitulate the complex interplay of different cell types of *in vivo* brain circuitry (see reviews [134–138]).

Another important decision for a biological follow up experiment is what type of genetic findings to model. Large-scale SZ case-control transcriptomic analysis has not been feasible due to the difficulties in scaling up the iPSC culture work and neuronal differentiation. The most straightforward experiments using hiPSC models have focused on studying cells from SZ patients carrying genetic risk variants of relatively large effect such as CNVs or rare variants [118,119]. The first hiPSC model of rare SZ risk variant is an isogenic model of frameshift mutation of disrupted in SZ 1 (DISC1). Forebrain cortical glutamatergic neurons derived from patent iPSCs carrying the DISC1 mutation showed reduced glutamatergic synapses and presynaptic transmission deficits, accompanying with dysfunctional expression changes of many genes related to synapses and psychiatric disorders [139]. As for SZ-associated CNVs, only two have been modeled in iPSCs: iPSCs carrying 15q11.2 (BP1-BP2) deletion showed abnormal neurogenesis [140] and dendritic morphology [141], and iPSC-derived neurons with 22q11.2 del recapitulated the miRNA expression patterns expected for 22q11.2 haploinsufficiency [142] and exhibited reduced neurosphere size, neural differentiation efficiency, neurite outgrowth, cellular migration and the neurogenic-to-gliogenic competence ratio [143]. Studying common SZ risk variants can be challenging, largely due to their small effects and variable genetic background between iPSC lines. However, some common risk variants of mental disorders have exhibited detectable biological effects on gene expression or cellular phenotypes in iPSC models. For instance, a GWAS risk variant (rs9834970) for bipolar disorder reduces the expression of its adjacent gene (*TRANK1*) in iPSC-derived neural cells, which can be rescued by chronic treatment with therapeutic dosages of valproic acid (VPA) [144]. With hiPSC-derived excitatory neurons combined with Clustered Regularly Interspaced Short Palindromic Repeats (CRISPR)/CRISPR-associated (Cas) nuclease-mediated genome editing, we also demonstrated that editing a common SZ GWAS SNP, rs1198588, at the *MIR137* locus resulted in detectable changes of chromatin accessibility of the noncoding *MIR137* promoter, *MIR137* expression, and neuronal phenotype [114].

To overcome possible confounding effects of variable genetic backgrounds when comparing differences between cells carrying risk versus non-risk alleles, a powerful strategy (such as we employed with *MIR137*) is to use CRISPR/Cas9 editing to generate isogenic iPSC-neurons, differing only at a single SNP site or a specific DNA sequence of interest [145–153]. Moreover, the applications of CRISPR/Cas9 technology in the past few years have evolved rapidly (see review [154]). In addition to changing a specific DNA sequence segment (e.g., insertion or deletion) or a SNP site, CRISPR/Cas9 can also epigenetically alter chromatin modification (e.g., histone methylation) without changing genomic DNA sequences (*i.e*., “CRISPR interference” or CRISPRi for transcriptional repression, and “CRISPR activation” or CRISPRa for transcriptional activation) [154]. In CRISPRi, deactivated Cas9 nuclease (dCas9), which does not break DNA, is fused with a Kruppel-associated box (KRAB) effector domain to achieve transcriptional repression by spreading repressive histone modifications, such as H3K9me3. For CRISPRa, dCas9 is fused to a DNA demethylase (e.g., TET1), a histone acetyltransferase (e.g., p300), or single activation effectors such as VP64 [154] to achieve targeted transcriptional activation. These approaches enable epigenomic perturbation of a regulatory genomic region around a putative disease causal variant, resulting in transcriptional repression or activation of its target gene(s). The most recent breakthrough gene editing technology, DNA base-editor, can covert A:T to G:C with very high efficiency (~50%), without creating a double strand DNA break and producing extremely low DNA off-target modifications [155], offering a powerful way to precisely change disease risk alleles in high efficiency.

A number of limitations remain when applying iPSC models to exploring disease biology of SZ and other mental disorders. Conceptually, the challenges include: (1) most common GWAS SNPs and rare risk variants (non-CNV) identified by exome sequencing have small effect sizes, which inhibits detection of meaningful biological effects; (2) the uncertainty of the putatively functional “causal” variants/genes at most GWAS risk loci may mask the true biological effects associated with disease risk; (3) most risk variants/genes do not function individually but rather interactively in a gene network, which implies the genetic perturbation of single variant/gene may not suffice; and (4) the widespread pleiotropic effects of disease risk loci may make it difficult to interpret the phenotypic outcomes from biological follow-up experiments. There are a number of technical challenges as well. First, hiPSC-derived neurons or cell types in brain organoids are relatively immature, representing the prenatal early neurodevelopmental stages. Second, there has been a lack of high-throughput neuronal phenotyping methods, hindering the use of these models to assay various cellular phenotypes for a large number of genetic variants. Third, although the 2D neuronal culture is relatively pure and thus more reproducible, phenotypic variability between different iPSC lines even from the same individual may compromise the sensitivity to detect small functional effects. Finally, although brain organoids as a model have been shown to achieve reasonable reproducibility in terms of type of neuronal cells in different organoids, the percentage of each cell type between organoids and between individuals remains quite variable [156]. Nonetheless, the rapidly improving methods to derive hiPSC models, in combination with CRISPR-based genome/epigenome perturbation, will help not only better understand the disease biology of SZ, but also facilitate the development of novel therapeutic approaches.

## CONCLUSION AND PERSPECTIVES

SZ is a complex disorder, involving both genetic and non-genetic factors. The understanding of SZ genetics has been a long journey characterized by waves of conceptual changes. With progress from a decade of GWAS, the field has finally reached a stage where abundant replicable genetic findings of SZ enable deeper exploration of SZ disease biology. Together, the interplay of genetic and non-genetic risk factors determines the disease phenotype through various biological mechanisms that may converge on some specific gene pathways.

Abnormal chromatin modifications represent a set of newly emerging mechanistic disease hypotheses. However, it remains elusive how chromatin remodeling affects disease phenotypes of SZ. Chromatin signaling may serve as a pivotal point for mediating the effects of both genetic and non-genetic SZ risk factors, which may, in part, explain the high clinical heterogeneity of SZ and the substantial pleiotropy of many of its risk variants. Finally, it is noteworthy that, while chromatin structure and its modifications are affected by SZ genetic factors, they also contribute to regional DNA mutation rates [157]. In this regard, given the prevalent somatic mutations (or mosaicism) in single neurons during human brain development and aging [158,159], it would be interesting to examine the interplay between chromatin signaling and the accumulation of somatic mutations in human brain development, and its relevance to SZ and other neurodevelopmental disorders. Nonetheless, given the known link between abnormal chromatin modifications and SZ, and the reversible nature of chromatin modifications, identifying small molecules that can perturb the chromatin states of core SZ gene networks in a tissue-specific manner may aid in the development of novel therapeutic interventions.

Rapidly evolving new technologies and approaches, such as hiPSC models and CRISPR-based genome/epigenome editing, have started to make inroads into understanding the disease biology of neurodevelopmental disorders. However, it remains to be seen how hiPSC models, including the emerging hiPSC-based brain organoids, in combination with CRISPR technology, can help to close the gap between genetic findings and clinical treatments of SZ. Moreover, defining specific functional readouts of disease phenotype and developing high-throughput screens of drug candidates (small chemicals or RNAs) for brain disorders like SZ, remains challenging. It is encouraging that new approaches such as genetics-led drug target prioritization (the priority index) are showing promise in integrating gene network/pathway analyses of GWAS findings with available high-throughput cellular screens data to predict effective drug targets for an array of immune disorders [160]. In this regard, single-cell technologies, e.g., transcriptomic profiles as a readout for pooled CRISPR screens, or other functional readouts such as single-cell Hi-C or ATAC-seq [154] applied to neurons, may help unravel SZ pathogenesis and develop novel therapeutics of SZ.
